# Huoshanmycins A‒C, New Polyketide Dimers Produced by Endophytic *Streptomyces* sp. HS-3-L-1 From *Dendrobium huoshanense*

**DOI:** 10.3389/fchem.2021.807508

**Published:** 2022-02-14

**Authors:** Youjuan Zhu, Yichao Kong, Yu Hong, Ling Zhang, Simin Li, Shurong Hou, Xiabin Chen, Tian Xie, Yang Hu, Xiachang Wang

**Affiliations:** ^1^ Jiangsu Key Laboratory for Functional Substances of Chinese Medicine, Nanjing University of Chinese Medicine, Nanjing, China; ^2^ Key Laboratory of Element Class Anti-Cancer Chinese Medicines, Engineering Laboratory of Development and Application of Traditional Chinese Medicines, Collaborative Innovation Center of Traditional Chinese Medicines of Zhejiang Province, School of Pharmacy, Hangzhou Normal University, Hangzhou, China

**Keywords:** huoshanmycin, polyketide, *Streptomyces*, *Dendrobium huoshanense*, endophyte

## Abstract

Three new polyketide dimers named huoshanmycins A‒C (**1**–**3**) were isolated from a plant endophytic *Streptomyces* sp. HS-3-L-1 in the leaf of *Dendrobium huoshanense*, which was collected from the Cultivation base in Jiuxianzun Huoshanshihu Co., Ltd. The dimeric structures of huoshanmycins were composed of unusual polyketides SEK43, SEK15, or UWM4, with a unique methylene linkage. Their structures were elucidated through comprehensive 1D-/2D-NMR and HRESIMS spectroscopic data analysis. The cytotoxicity against MV4-11 human leukemia cell by the Cell Counting Kit-8 (CCK8) method was evaluated using isolated compounds with triptolide as positive control (IC_50_: 1.1 ± 0.4 μM). Huoshanmycins A and B (**1**, **2**) displayed moderate cytotoxicity with IC_50_ values of 32.9 ± 7.2 and 33.2 ± 6.1 μM, respectively.

## Introduction

*Dendrobium huoshanense* is a perennial epiphytic Orchidaceae herb with important medicinal and ornamental value. The leaves of *D. huoshanense* have long been utilized for dermatologic disorders, metabolic syndromes, nervous system disorders, and musculoskeletal system disorders (Wang, 2021). Modern pharmacological research has revealed that *D. huoshanense* has anti-inflammatory (Ge et al., 2018; Li et al., 2020), cytotoxic (Chen et al., 2022), hypoglycemic (Wang et al., 2019), anti-atherosclerosis (Fan et al., 2020), and antioxidant (Tian et al., 2013) activity. *Dendrobium* plant is well-known for its rich and diverse endophytic bacterial and fungal community (Chen et al., 2019; Chen et al., 2020). Previous studies have revealed the close relationship between *Dendrobium* and its endophytes, such as improving the seed germination rate (Tsavkelova et al., 2007) and supply of nutrients (Li et al., 2017). At present, research on *D. huoshanense* endophytes mainly focuses on the diversity and functions, while not much is known about their secondary metabolites, especially for bacteria. The main species of bacterial microorganisms of *D. huoshanense* are *Sphingomonas, Acinetobacter, Enterococcus, Bacillus*, and *Methylobacterium* (Chen et al., 2020). *Streptomyces* is the largest genus of Actinobacteria and characterized by producing complex secondary metabolites. They produce over two-thirds of the clinically useful antibiotics of natural origin (e.g., chloramphenicol, streptomycin, tetracycline, erythromycin, ivermectin, and rifamycin) (Raja and Prabakarana, 2011). The last four compounds all belong to polyketides, which are derived from a precursor molecule consisting of a chain of alternating ketone (or reduced forms of a ketone) and methylene groups.

Since the discovery of taxol and taxane produced by an endophytic fungus from the phloem (inner bark) of Pacific yew in 1993 (Stierle et al., 1993), endophytes have become an important resource in the field of bioactive natural products discovery (Newman and Cragg, 2015; Gómez and Luiz, 2018), as they can produce analogs or bioactivity-related compounds as their hosts did (Cui et al., 2012; Zhao et al., 2020c). As part of an effort to characterize novel natural products from medicinal plants (Wang et al., 2009; Ding et al., 2021; Hu et al., 2021) and their endophytes (Zhao et al., 2020b; Zhao et al., 2020a; Zhu et al., 2021), herein we report the isolation and characterization of three new polyketide dimers from an endophyte *Streptomyces* sp. HS-3-L-1 of the *D. huoshanense* leaf. The dimeric structures of new huoshanmycins A‒C (**1**–**3**) were composed of SEK43, SEK15, or UWM4 (Meurer et al., 1997), with a unique methylene linkage. Herein, we report the fermentation, extraction, isolation, structural elucidation, and cytotoxic activity of these secondary metabolites.

## Materials and Methods

### General Experimental Procedures

UV data were acquired on a Persee TU-1810 spectrophotometer (Persee analytics, Beijing, China). IR spectra were measured on a Thermo Scientific Nicolet iS5 FT-IR spectrometer (Thermo, United States). NMR spectra were obtained on a Bruker Advance AV500 spectrometer (Bruker, Germany). HRESIMS spectra were recorded on an Orbitrap Elite mass spectrometer (Thermo Scientific, United States). Liquid chromatography–mass spectrometry (LC-MS) was conducted with an Agilent 1290 system equipped with 6120 Quadrupole MSD mass spectrometer (Agilent Technologies, United States). HPLC analysis was performed on a Waters 2695 system equipped with 2998 PDA detector. Total component analysis was performed on an Agilent 1290 UHPLC-6520 Q-TOF/MS. Preparative HPLC separation was performed on a Waters 1525 EF LC system (Waters Company, United States). MCI GEL high-porous polymer (75–150 μm) was purchased from Mitsubishi Chemical Corporation (Japan). Sephadex LH-20 resin (25–100 μm) was purchased from GE Healthcare Company (Sweden). XAD16N resin (20–60 mesh) was obtained from Yuanye Company (Nanjing, Jiangsu, China). Chemicals were purchased from Juyou Company or Aldrich and used without further purification unless otherwise noted.

### Strain Isolation

Plant samples of *D. huoshanense* were provided by Jiuxianzun Huoshanshihu Co., Ltd. (Liu-An City, Anhui Province, China) and identified by co-author Dr. Yang Hu. A voucher specimen (no. 20190309) was deposited at Jiangsu Key Laboratory for Functional Substances of Chinese Medicine, China. The roots, leaves, and stems of *D. huoshanense* were separated and cleaned with water and then rinsed in 0.1% Tween-20 for 30 s, sequentially immersed in 75% ethanol for 5 min and in 2% sodium hypochlorite for 5 min and rinsed with 10% NaHCO_3_ for 10 min to inhibit fungal growth. After each treatment, samples were rinsed three times in sterile water. The surface sterilized samples were aseptically dissected into small pieces; 0.5 g of each sample was suspended in 1.0 ml of sterile H_2_O, and heated at 75°C for 1 min to eliminate nonsporulating bacteria (Zhao et al., 2020a). A 100-μl aliquot of supernatant was streaked on oatmeal agar and on ISP4 agar plates supplemented with nalidixic acid (25 μg/ml) and amphotericin B (25 μg/ml). A number of sporulating bacterial colonies were observed after 1–2 months of incubation at 28°C, and each colony was subsequently purified on a M2 agar plate (Wang et al., 2013). Overall, 54 endophytic strains were isolated from plant samples. The endophytic strain HS-3-L-1 was isolated from the leaf of *D. huoshanense.*

### Phylogenetic Analysis

Strain HS-3-L-1 was inoculated in a 20-ml test tube with 4 ml of TSB broth. After 3 days culture at 28°C with 160 rpm agitation, the partial 16S rRNA gene fragment was amplified using universal primers (27F 5′-AGAGTTTGATCMTGGCTCAG-3’; 1492R 5′-GGT​TAC​CTT​GTT​ACG​ACT​T-3′). The amplified fragment (1,367 bp) was sent for sequencing analysis (Shanghai Sangong Company, China), which displayed 99.85% identity (BlastN, https://blast.ncbi.nlm.nih.gov/Blast.cgi) to *Streptomyces polaris* (MW164959.1). The sequence of 16S rRNA has been deposited in the NCBI nucleotide database with the accession number OK161010.

### Fermentation, Extraction, and Isolation

*Streptomyces* sp. HS-3-L-1 was grown on M2 agar plate (glucose 4 g/L, malt extract 10 g/L, yeast extract 4 g/L, and agar 15 g/L) at 28°C for a week. Small pieces of agar with bacterial growth were added to eleven 250-ml Erlenmeyer flasks, each containing 50 ml of medium Bran [corn flour, 40.0 g/L; gluten powder, 5.0 g/L; K_2_HPO_4_•3H_2_O, 0.5 g/L; glucose, 10.0 g/L; bran, 10.0 g/L; CaCO_3_, 2.0 g/L; and (NH_4_)_2_SO_4_, 1.0 g/L]. After 3 days of incubation at 28°C with 200 rpm agitation, the seed cultures were used to inoculate 100 Erlenmeyer flasks (250 ml), each containing 100 ml of medium Bran (total 10 L). The fermentation was carried out on a rotary shaker (200 rpm) at 28°C for a week. All obtained culture broth was combined and centrifuged at 5,000 ×*g* for 30 min to separate the mycelium and supernatant. Mycelium was extracted with MeOH (3 × 2 L), and the organic phase was evaporated to afford 50.2 g of crude extract A. The supernatant was mixed with 4% (w/v) XAD-16 resin and stirred for 6 h, followed by filtration. The resin was washed with water (3 × 500 ml) and then eluted with MeOH until the eluant was colorless. The MeOH extract was subsequently evaporated to afford 13.3 g of crude extract B.

Crude extract A (50.2 g) was subjected to an MCI column (500 g, 10 × 80 cm) and eluted with a gradient of aqueous MeOH (20, 40, 60, 80, and 100%) to yield 16 fractions (Fr. 1-1 to Fr. 1-16). Fr. 1-9 (0.5 g) was subjected to a Sephadex LH-20 column (4 × 100 cm, 2 ml/min) eluted with 80% aqueous MeOH to obtain 11 subfractions (Fr. 1-9-1 to Fr. 1-9-11). Compound **5** (13.6 mg) was obtained from Fr. 1-9-4. Fr. 1-9-9 was further purified by semi-preparative HPLC (38% aqueous MeOH) to yield compound **3** (5.8 mg). Crude extract B (13.3 g) was subjected to an MCI column (200 g, 6 × 30 cm), using gradient elution from 20% to 100% aqueous MeOH to provide 11 fractions (Fr. 2-1 to Fr. 2–11). Fractions Fr. 2-5 (0.7 g) was subjected to a Sephadex LH-20 column (4 × 100 cm, 2 ml/min, 80% aqueous MeOH) to obtain 12 subfractions (Fr. 2-5-1 to Fr. 2-5-12). Fr. 2-5-6 (0.3 g) was further purified by semi-preparative HPLC (45% aqueous MeOH) to yield compounds **4** (7.6 mg) and **7** (9.4 mg). Fr. 2-10 (0.5 g) was also purified by a Sephadex LH-20 column (4 × 100 cm, 2 ml/min, 80% aqueous MeOH) to obtain seven subfractions (Fr. 2-10-1 to Fr. 2-10-7). Fr. 2-10-6 (0.2 g) was further purified by semi-preparative HPLC (63% aqueous MeOH) to yield compounds **1** (8.6 mg), **2** (16.8 mg), and **6** (12.3 mg).

Huoshanmycin A (**1**): Yellow amorphous powder; UV (MeOH) λ_max_ (log *ε*) 294 nm (4.17); IR (KBr) *ν*_max_ 3,164, 2,927, 1,680, 1,617, 1,437, 1,401, 1,384, 1,284, 1,207, 1,138, 1,027, 829, 803, and 724 cm^−1^; ^13^C and ^1^H NMR data, see Table 1; (+)-ESI-MS: *m/z* 749.0 [M + H]^+^; (‒)-ESI-MS: *m/z* 747.2 [M − H]^‒^; (−)-HR-ESI-MS *m/z* 747.17096 [M − H]^−^ (calcd. for C_41_H_31_O_14_ 747.1714).

**TABLE 1:** ^1^H (500 MHz) and ^13^C (125 MHz) NMR data of compounds **1**‒**3** in DMSO-*d*_6_.

No	1		2		3	
	*δ*_C_, type	*δ*_H_, *J* in Hz	*δ*_C_, type	*δ*_H_, *J* in Hz	*δ*_C_, type	*δ*_H_, *J* in Hz
1	165.2, C	—	165.2, C	—	165.2, C	—
2	100.0, C	—	100.1, C	—	100.1, C	—
3	166.1, C	—	166.1, C	—	166.3, C	—
4	101.8, CH	5.76, s	101.8, CH	5.75, s	101.6, CH	5.74, s
5	160.9, C	—	160.9, C	—	160.8, C	—
6	36.5, CH_2_	3.54, s	36.6, CH_2_	3.55, s	36.5, CH_2_	3.53, s
7	133.2, C	—	133.2, C	—	133.2, C	—
8	121.3, CH	6.74, dd (7.6, 2.1)	121.3, CH	6.75, d (7.6)	121.2, CH	6.75, d (7.7)
9	130.5, CH	7.22, t (7.7)	130.5, CH	7.22, t (7.9)	130.5, CH	7.21, t (7.9)
10	115.0, CH	6.80, t (8.7)	115.0, CH	6.80, d (8.2)	114.9, CH	6.80, d (8.2)
11	154.2, C	—	154.2, C	—	154.2, C	—
12	131.2, C	—	131.3, C	—	131.2, C	—
13	200.4, C	—	200.4, C	—	200.6, C	—
14	116.0, C	—	116.0, C	—	116.0, C	—
15	165.5, C	—	165.5, C	—	165.5, C	—
16	101.1, CH	6.13, d (2.3)	101.8, CH	6.14, s	101.1, CH	6.13, d (2.1)
17	163.7, C	—	163.6, C	—	163.6, C	—
18	112.0, CH	6.09, d (2.0)	112.0, CH	6.09, s	112.0, CH	6.09, d (1.7)
19	143.4, C		143.4, C		143.4, C	
20	21.9, CH_3_	1.83, s	21.9, CH_3_	1.84, s	21.9, CH_3_	1.84, s
1′	161.9, C	—	165.2, C	—	165.1, C	—
2′	100.1, C	—	100.1, C	—	100.0, C	—
3′	166.3, C	—	166.1, C	—	166.2, C	—
4′	101.7, CH	5.70, s	101.8, CH	5.75, s	101.5, CH	5.74, s
5′	161.4, C	—	160.9, C	—	161.2, C	—
6′	37.0, CH_2_	3.47, s	36.6, CH_2_	3.55, s	36.7, CH_2_	3.48, s
7′	134.8, C	—	133.2, C	—	135.8, C	—
8′	120.9, CH	6.74, dd (7.6, 2.1)	121.3, CH	6.75, d (7.6)	109.5, CH	6.16, d (1.9)
9′	129.1, CH	7.17, t (7.9)	130.5, CH	7.22, t (7.9)	160.1, C	
10′	114.4, CH	6.80, t (8.7)	115.0, CH	6.80, d (8.2)	101.9, CH	6.23, d (1.9)
11′	154.7, C	—	154.2, C	—	157.8, C	—
12′	129.6, C	—	131.3, C	—	121.5, C	—
13′	141.1, C	—	200.4, C	—	200.3, C	—
14′	111.3, CH	5.95, d (2.4)	116.0, C	—	117.6, C	—
15′	162.4, C	—	165.5, C	—	163.2, C	—
16′	102.3, CH	6.27, d (2.4)	101.8, CH	6.14, s	100.9, CH	6.11, s
17′	163.6, C	—	163.6, C	—	162.4, C	—
18′	115.9, C	—	112.0, CH	6.09, s	111.3, CH	6.06, s
19′	203.6, C	—	143.4, C	—	142.1, C	—
20′	30.3, CH_3_	1.87, s	21.9, CH_3_	1.84, s	21.4, CH_3_	1.83, s
1″	17.8, CH_2_	3.25, s	17.8, CH_2_	3.24, s	17.7, CH_2_	3.24, s
11-OH	—	9.81, s	—	9.82, s	—	9.83, s
15-OH	—	12.66, s	—	12.68, s	—	12.68, s
17-OH	—	10.44, s	—	10.45, s	—	10.45, s
9′-OH	—	—	—	—	—	9.88, s
11′-OH	—	9.48, s	—	9.82, s	—	10.26, s
15′-OH	—	10.36, s	—	12.68, s	—	12.05, s
17′-OH	—	12.50, s	—	10.45, s	—	10.21, s

Huoshanmycin B (**2**): Yellow amorphous powder; UV (MeOH) λ_max_ (log *ε*) 294 nm (4.44); IR (KBr) *ν*_max_ 3,184, 2,260, 2,129, 1,675, 1,587, 1,464, 1,284, 1,167, 1,158, 1,024, 995, 926, 826, 769, 722, 699, 656, 633, 611, 576, and 523 cm^−1^; ^13^C and ^1^H NMR data, see Table 1; (+)-ESI-MS: *m/z* 749.0 [M + H]^+^; (‒)-ESI-MS: *m/z* 747.2 [M − H]^‒^; (+)-HR-ESI-MS *m/z* 749.18768 [M + H]^+^ (calcd. for C_41_H_33_O_14_ 749.1970).

Huoshanmycin C (**3**): Yellow amorphous powder; UV (MeOH) λ_max_ (log *ε*) 294 nm (4.41); IR (KBr) *ν*_max_ 3,172, 2,259, 2,129, 1,677, 1,616, 1,587, 1,464, 1,384, 1,271, 1,204, 1,169, 1,142, 1,024, 999, 926, 844, 800, 766, 722, 600, and 524 cm^−1^; ^13^C and ^1^H NMR data, see Table 1; (+)-ESI-MS: *m/z* 765.2 [M + H]^+^; (‒)-ESI-MS: *m/z* 763.2 [M − H]^‒^; (+)-HR-ESI-MS *m/z* 765.1819 [M + H]^+^ (calcd. for C_41_H_33_O_15_, 765.1819), 787.1640 [M + Na]^+^ (calcd. for C_41_H_32_O_15_Na, 787.1639).

### Cell Culture and Proliferation Inhibition Assay

The human AML cell line MV4-11 (CRL-9591) was purchased from ATCC and cultured in IMDM (Gibco) supplemented with 10% FBS (Gibco) and 1% penicillin–streptomycin (Gibco). To conduct cell proliferation assay, cells (1.5 × 10^6^ cells/well) in the logarithmic phase were seeded into 96-well plates simultaneously with various concentrations of different compounds (5 μl, final concentration of 50–0.023 µM for IC_50_ determination) or vehicle (0.5% DMSO) for 48 h. Cell viability of compounds **1**‒**4** was measured using Cell Counting Kit-8 (DoJINDO) according to the manufacturer’s instructions with triptolide as positive control (Table 2). The absorbance was measured at 450 nm using a microplate reader (Epoch, Bio-Tek, United States). The value of half maximal inhibitory concentration (IC_50_) was calculated using GraphPad Prism 7.

**TABLE 2:** Antiproliferative activity of compounds **1**‒**4** against MV4-11 cell line.

Compounds	IC_50_ ± SD, μM
**1**	32.9 ± 7.2
**2**	33.2 ± 6.1
**3**	>50
**4**	>50
Triptolide	1.1 ± 0.4

### Antimicrobial Assay

Standard strains of *Staphylococcus aureus* (ATCC 29213), *Escherichia coli* (ATCC 25922), *Bacillus subtilis* (A186), *Pseudomonas aeruginosa* (ATCC 27853), and *Acinetobacter baumannii* (ATCC 19606) were obtained from CICC (China Center of Industrial Culture Collection, China). Bacteria were inoculated in LB Broth media and incubated overnight at 37°C. The cultures were quantified *via* a spectrophotometer, and then diluted to A = 0.02 (OD_600_) and dispensed to 96-well black, clear-bottom assay plates (100 µl/well). Test compounds (final concentration 50 µM) and controls (positive control of 50 µM polymyxin and placebo control of DMSO) were then added. The plates were incubated for 16 h at 37°C, and then measured the absorbance at 600 nm using a microplate reader. Compound activity was calculated on a per-plate basis (Zhao et al., 2020a).

## Results and Discussion

**FIGURE 1:**
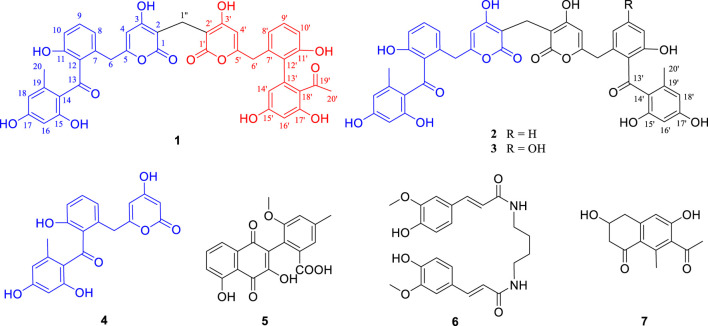
Chemical structures of compounds isolated from *Streptomyces* sp. HS-3-L-1.

Preliminary HPLC-HRMS metabolic profiling of endophytic actinomycete strains isolated from *D. huoshanense* plant samples revealed that *Streptomyces* sp. HS-3-L-1 is capable of novel secondary metabolite production (Supplementary Figure S1). After scale-up fermentation (10 L) of *Streptomyces* sp. HS-3-L-1, and further extraction, fractionation, and standard chromatography, three new polyketide dimers were isolated and identified [huoshanmycins A (**1**, yield: 1.68 mg/L), B (**2**, yield: 0.86 mg/L), and C (**3**, yield: 0.58 mg/L)], together with four previously reported metabolites, SEK43 (**4**, yield: 0.76 mg/L), WS-5995 C (**5**, yield: 1.36 mg/L), JBIR-94 (**6**, yield: 12.3 mg/L), and GTRI-02 (**7**, yield: 0.94 mg/L)] (Figure 1).

**FIGURE 2:**
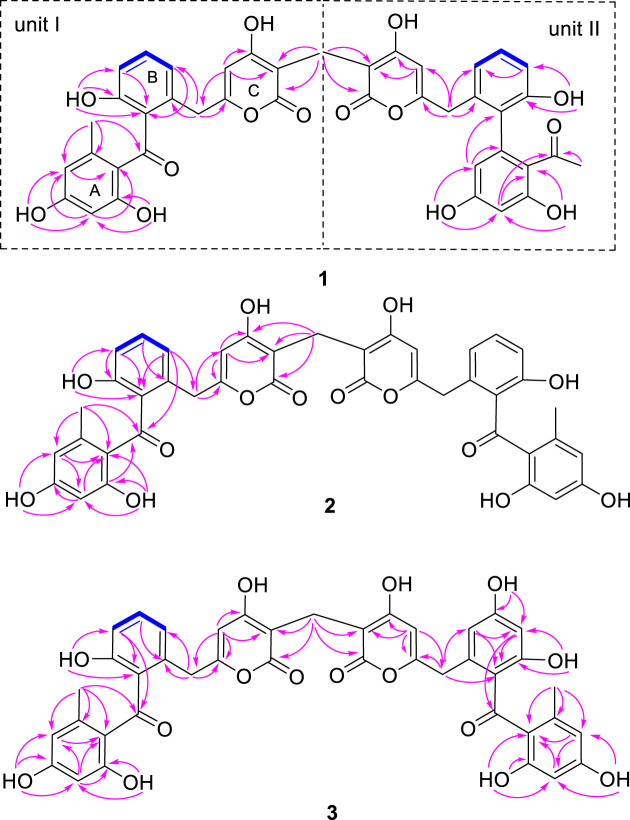
Key COSY (bolds, blue), HMBC (arrows, pink) correlations of 1**‒**3.

Compound **1** was obtained as a yellow amorphous powder. Its molecular formula was established as C_41_H_32_O_14_ based on HRESIMS data, which indicated 26 degrees of unsaturation. The ^13^C NMR of compound **1** showed 41 carbons, which can be sorted into two methyls, three methylenes, twelve aromatic methines, twenty sp^2^ quarternary carbons, and four carbonyls/carboxylic acids, with the aid of HSQC spectrum. The ^1^H-NMR and HSQC spectra of **1** indicated twelve aromatic protons [*δ*_H_ 5.70 (1H, s), 5.76 (1H, s), 5.95 (1H, d, *J* = 2.4 Hz), 6.09 (1H, d, *J* = 2.0 Hz), 6.13 (1H, d, *J* = 2.3 Hz), 6.27 (1H, d, *J* = 2.4 Hz), 6.74 (2H, dd, *J* = 2.1, 7.6 Hz), 6.80 (2H, t, *J* = 8.7 Hz), 7.17 (1H, t, *J* = 7.9 Hz), and 7.22 (1H, t, *J* = 7.7 Hz)], two methyls [*δ*_H_ 1.83 and 1.87 (each 3H, s)], three methylenes [*δ*_H_ 3.25, 3.47, and 3.54 (each 2H, s)], and six hydroxyls showed at low field: *δ*_H_ 9.48, 9.81, 10.36, 10.44, 12.50, and 12.66 (each 1H, s). Two spin systems of H-8 (*δ*_H_ 6.74)/H-9 (*δ*_H_ 7.22)/H-10 (*δ*_H_ 6.80) and H-8′ (*δ*_H_ 6.74)/H-9′ (*δ*_H_ 7.17)/H-10′ (*δ*_H_ 6.80) observed in COSY spectrum suggested the presence of two 1,2,3-trisubstiuted benzene rings. Two aromatic protons *δ*_H_ 6.13 (H-16) and 6.09 (H-18), together with the HMBC correlations from H_3_-20 (*δ*_H_ 1.83) to C-13 (*δ*_C_ 200.4), C-18 (*δ*_C_ 112.0), and C-19 (*δ*_C_ 143.4), and from H-16 to C-14 (*δ*_C_ 116.0) and C-18, and from H-18 to C-14, C-16 (*δ*_C_ 101.1), and C-20 (*δ*_C_ 21.9) suggested the presence of a 2,4-dihydroxy-6-methyl-1-keto-phenyl moiety (ring A in Figure 2). The two active hydrogens showed key HMBC correlations from 15-OH (*δ*_H_ 12.66, s) to C-14/C-15/C-16, and from 17-OH (*δ*_H_ 10.44, s) to C-16/C-17/C-18, indicating two hydroxyl groups located at C-15 and C-17. An unsaturated six-member lactone ring (ring C in Figure 2) was constructed by HMBC cross peaks from H-4 (*δ*_H_ 5.76) to C-2 (*δ*_C_ 100.0), C-3 (*δ*_C_ 166.1), and C-6 (*δ*_C_ 36.5), and from H-1″ to C-1 (*δ*_C_ 165.2) and C-2. The obvious HMBC correlations from H_2_ -6 (*δ*_H_ 3.54) to C-4 (*δ*_C_ 101.8), C-5 (*δ*_C_ 160.9), C-7 (*δ*_C_ 133.2) and C-8 (*δ*_C_ 121.3) permitted the assembly of ring B and ring C through a CH_2_ linkage. Analysis of the remaining NMR data revealed that unit I of **1** (Figure 2) as C2-substitued SEK43 (**4**) (Meurer et al., 1997). SEK43 was reported as an engineered biosynthesis product, which was isolated and identified likewise from this endophytic strain. In a similar manner, the other decaketide-related subunit II was elucidated as C2′-substitued UMW4 (Meurer et al., 1997) mainly through HMBC correlations (Figure 2), e.g., from H-4′ (*δ*_H_ 5.70, s) to C-2′ (*δ*_C_ 100.1) and C-3′ (*δ*_C_ 166.3), from H_2_-6′ (*δ*_H_ 3.47, s) to C-4′, C-5′ (*δ*_C_ 161.4), C-7′ (*δ*_C_ 134.8), and C-8′ (*δ*_C_ 120.9), from H-14′ (*δ*_H_ 5.95, d) to C-12′ (*δ*_C_ 129.6) and C-13′ (*δ*_C_ 141.1), and from H-16′ (*δ*_H_ 6.27, d) to C-14′ (*δ*_C_ 111.3), C-18′ (*δ*_C_ 115.9), and C-19′ (*δ*_C_ 203.6). The strong HMBC correlations from H_2_-1′′ (*δ*_H_ 3.25) to C-1 (*δ*_C_ 165.2), C-2 (*δ*_C_ 100.0), C-1′ (*δ*_C_ 161.9), and C-2′ (*δ*_C_ 100.1) unambiguously connected SEK43 and UMW4 with a unique methylene bridge, to form the final dimeric structure of **1**. Thus, compound **1** was identified as a novel polyketide dimer and named huoshanmycin A, to reflect the producing strain’s point of origin.

Compound **2** was also obtained as a white amorphous powder and shared the same molecular formula (C_41_H_32_O_14_) with huoshanmycin A (**1**). Compound **2** was clearly recognized as a polyketide dimer from its NMR data (Table 1), which showed only 21 carbons in ^13^C NMR spectrum. Analysis of NMR data of compound **2** revealed that it was highly similar to SEK43 (**4**) and shared a same methylene linkage between C-2 and C-2′. This was confirmed by HMBC correlations from H_2_-1" (*δ*_H_ 3.24) to C-1/C-1′ (*δ*_C_ 165.2), C-2/C-2′ (*δ*_C_ 100.1), and C-3/C-3′ (*δ*_C_ 166.1). The remaining HMBC correlations (Figure 2) and NMR data (Table 1) were in full agreement with the new structure of compound **2**, and it was named huoshanmycin B.

Compound **3** was obtained as a yellow amorphous powder and its molecular formula was determined as C_41_H_32_O_15_ from HRESIMS results. A detailed comparison of the NMR data of **3** and **2** indicated that their structures were highly similar. The significant differences observed in NMR spectra was that one of the 1,2,3-trisubstiuted benzene ring protons in **2** (*δ*_H_ 6.75, 7.22, 6.80) was replaced with two olefinic methines (*δ*_H_ 6.16, 6.23) and one hydroxyl (*δ*_H_ 9.88) in compound **3**. This tetra-substituted benzene moiety was confirmed by the HMBC correlations from H-6′ (*δ*_H_ 3.48) to C-7, C-8′, and C-12′, from H-8′ (*δ*_H_ 6.16) to C-6′, C-9′, C-10′, and C-12′, and from H-10′ (*δ*_H_ 6.23) to C-8′, C-9′, C-11′, and C-12′, as well as the correlation signals from 9′-OH (*δ*_H_ 9.88) to C-8′, C-9′, and C-10′. Therefore, the structure of huoshanmycin C (**3**) was elucidated as shown in Figure 1.

The other five known compounds were identified as SEK43 (**4**) (McDaniel et al., 1995), WS-5995 C (**5**) (Ikushima et al., 1983), JBIR-94 (**6**) (Taj and Sorensen, 2015), and GTRI-02 (**7**) (Yeo et al., 1998), through comparison with reported data. Although compounds **1**–**7** were inactive at or below 50 μM in a standard antimicrobial assay, huoshanmycins A and B (**1**, **2**), the two isomers, showed antiproliferative activity against the MV4-11 cell line with IC_50_ values of 32.9 ± 7.2 and 33.2 ± 6.1 μM, respectively (Table 2).

In summary, the discovery of compounds **1**–**7** as metabolites of the *D. huoshanense* isolate *Streptomyces* sp. HS-3-L-1 further highlights the potential for novel microbial natural product discovery from medicinal plants. Among them, compounds **1**–**3** were unique dimers of SEK43 (**4**), SEK15, or UWM4, three decaketide-related shunt products discovered from minimal *jad*PKS constructs (Meurer et al., 1997). So far, only two similar natural products, strepolyketides B and C, were recently reported from a marine-derived *Streptomyces* (Jiang et al., 2020). Moreover, huoshanmycins A and B showed moderate cytotoxicity against MV4-11 human leukemia cell. The newly isolated **1**–**3** enriched the structural diversity of microbial source. Future investigation to explore the biosynthetic logic of these structurally unique dimers is ongoing.

## Data Availability

The datasets presented in this study can be found in online repositories. The names of the repository/repositories and accession number(s) can be found below: https://www.ncbi.nlm.nih.gov/, OK161010.
