# Supplementary material for: Huoshanmycins A‒C, New Polyketide Dimers Produced by Endophytic Streptomyces sp. HS-3-L-1 From Dendrobium huoshanense
Source: Front Chem. 2022 Feb 14;9:807508. doi: 10.3389/fchem.2021.807508 (PMC8883461; doi:10.3389/fchem.2021.807508)
Supplement: Supplementary file 1 [file DataSheet1.pdf]

Supplementary Material

Huoshanmycins A–C, New Polyketide Dimers Produced by Endophytic *Streptomyces* sp. HS-3-L-1 from *Dendrobium huoshanense*

Youjuan Zhu[1,#], Yichao Kong[2,#],Yu Hong[1], Ling Zhang[1], Simin Li[1], Shurong Hou[2], Xiabin Chen[2], Tian Xie[2], Yang Hu[1*] and Xiachang Wang[1*]

CONTENT

Comprehensive structural characterization
Supplementary Figure 1. The representative MS/MS of HS-3-LEAF-1 crude extract. (A) Positive mode, (B) Negative mode, (C) MS/MS spectra and possible fragmentation patterns of compounds **2** and **3**.
Supplementary Figure 2. ^{1}H NMR spectrum of **1** (DMSO-d_6, 500 MHz).
Supplementary Figure 3. ^{13}C NMR spectrum of **1** (DMSO-d_6, 125 MHz).
Supplementary Figure 4. HSQC spectrum of **1** (DMSO-d_6, 500 MHz).
Supplementary Figure 5. HMBC spectrum of **1** (DMSO-d_6, 500 MHz).
Supplementary Figure 6. ^{1}H-^{1}H COSY spectrum of **1** (DMSO-d_6, 500 MHz).
Supplementary Figure 7. (+) and (–)-ESI-MS of **1**.
Supplementary Figure 8. HR-ESI-MS spectrum of **1**.
Supplementary Figure 9. ^{1}H NMR spectrum of **2** (DMSO-d_6, 500 MHz).
Supplementary Figure 10. ^{13}C NMR spectrum of **2** (DMSO-d_6, 125 MHz).
Supplementary Figure 11. HSQC spectrum of **2** (DMSO-d_6, 500 MHz).
Supplementary Figure 12. HMBC spectrum of **2** (DMSO-d_6, 500 MHz).
Supplementary Figure 13. ^{1}H-^{1}H COSY spectrum of **2** (DMSO-d_6, 500 MHz).
Supplementary Figure 14. (+) and (–)-ESI-MS of **2**.
Supplementary Figure 15. HR-ESI-MS spectrum of **2**.
Supplementary Figure 16. ^{1}H NMR spectrum of **3** (DMSO-d_6, 500 MHz).
Supplementary Figure 17. ^{13}C NMR spectrum of **3** (DMSO-d_6, 125 MHz).
Supplementary Figure 18. HSQC spectrum of **3** (DMSO-d_6, 500 MHz).
Supplementary Figure 19. HMBC spectrum of **3** (DMSO-d_6, 500 MHz).
Supplementary Figure 20. ^{1}H-^{1}H COSY spectrum of **3** (DMSO-d_6, 500 MHz).
Supplementary Figure 21. (+) and (–)-ESI-MS of **3**.
Supplementary Figure 22. HR-ESI-MS spectrum of **3**.

Comprehensive structural characterization

The Agilent 1290 UHPLC-6520 Q-TOF/MS system (Palo Alto, CA, USA) with an ACQUITY UHPLC HSS T3 column (150 mm × 2.1 mm, 1.8 µm) was applied for separation. The mobile phase consisted of water containing 0.1% formic acid (A) and methanol (B). The gradient elution was started from 30% B, then increased linearly to 100% B over 35 min, then maintained for 5 min. The flow rate was set at 0.2 mL/min. Data acquisition was progressed in both of positive and negative ionization modes (**Supplementary Figures 1A** and **1B**). Mass spectrometry conditions were set as follows: ion source temperature, 120°C; capillary voltage, 4.0 kV in positive ion mode and 3.5 kV in negative ion mode; mass scan range, m/z 50–1500 Da.

The analysis results show that some potential new compounds were detected: *m/z* 747.1713 $[M - H]^-$ (calcd. for $C_{41}H_{31}O_{14}$ 747.1714), *m/z* 763.1679 $[M - H]^-$ (calcd. for $C_{41}H_{31}O_{15}$ 763.1663) (**Supplementary Figure 1C**).

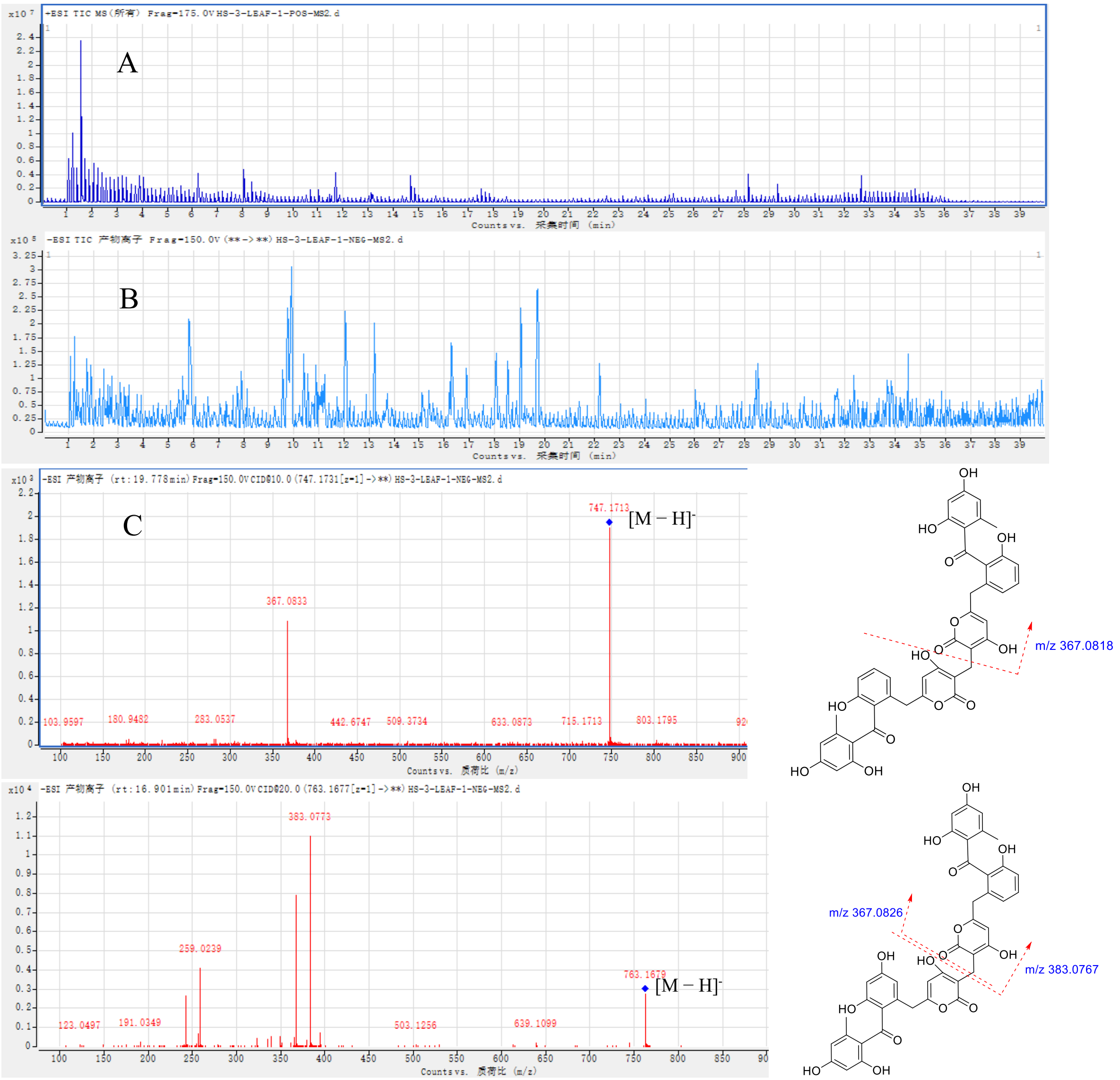

Supplementary Figure 1. The representative MS/MS of HS-3-LEAF-1 crude extract. (A) Positive mode, (B) Negative mode, (C) MS/MS spectra and possible fragmentation patterns of compounds **2** and **3**.

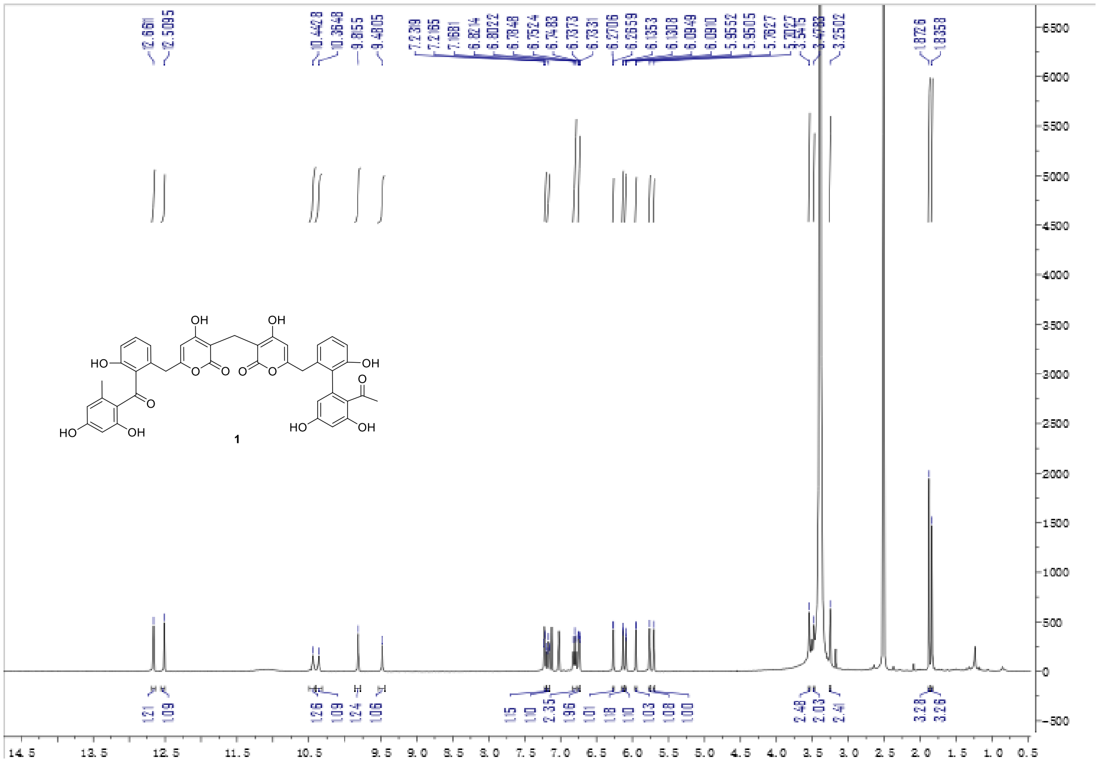

Supplementary Figure 2. ^{1}H NMR spectrum of **1** (DMSO-d_6, 500 MHz).

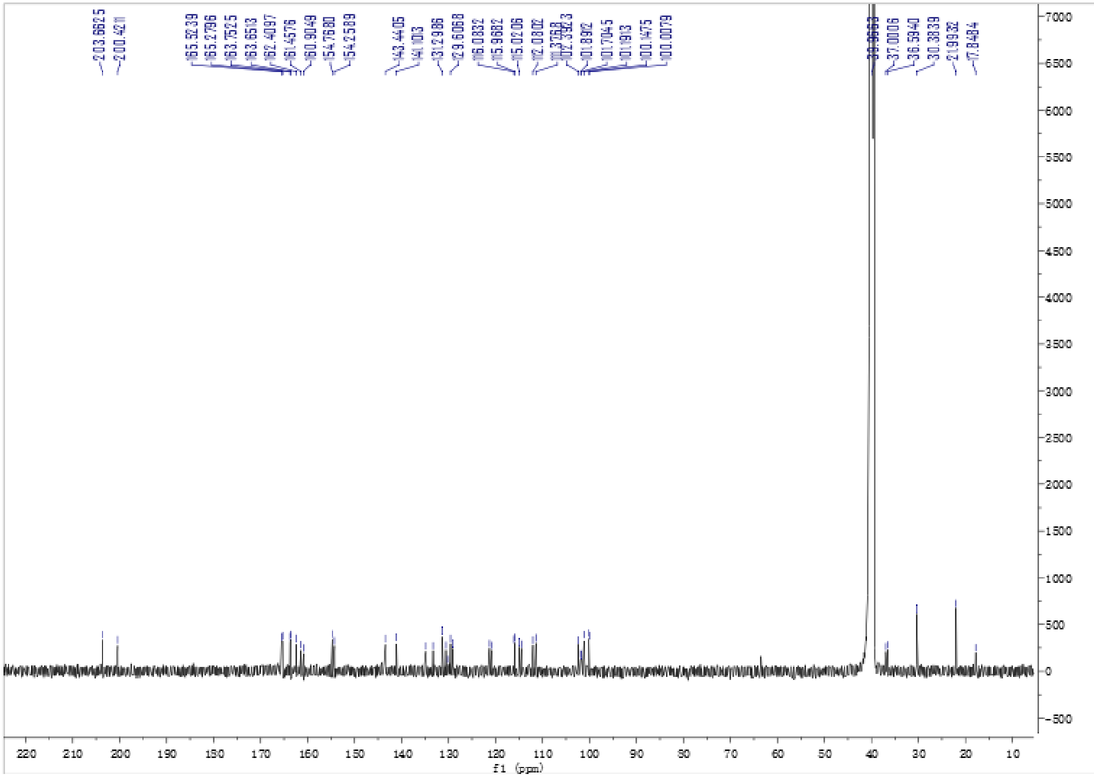

Supplementary Figure 3. ^{13}C NMR spectrum of **1** (DMSO-d_6, 125 MHz).

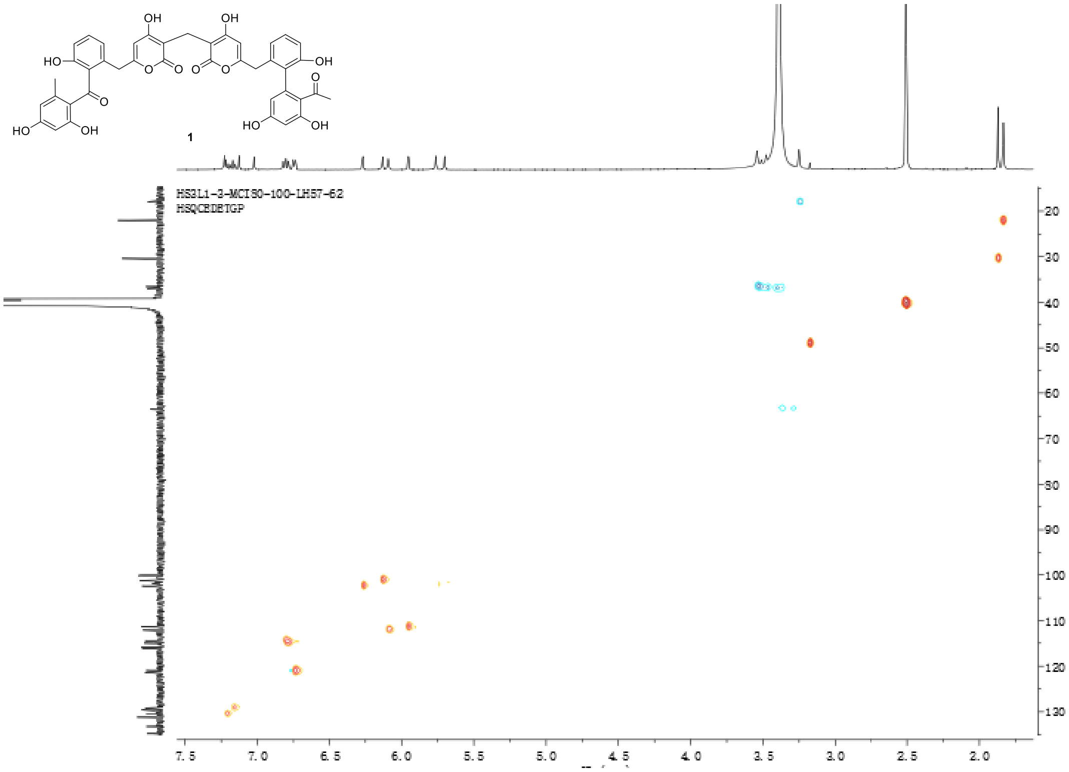

Supplementary Figure 4. HSQC spectrum of **1** (DMSO-d_6, 500 MHz).

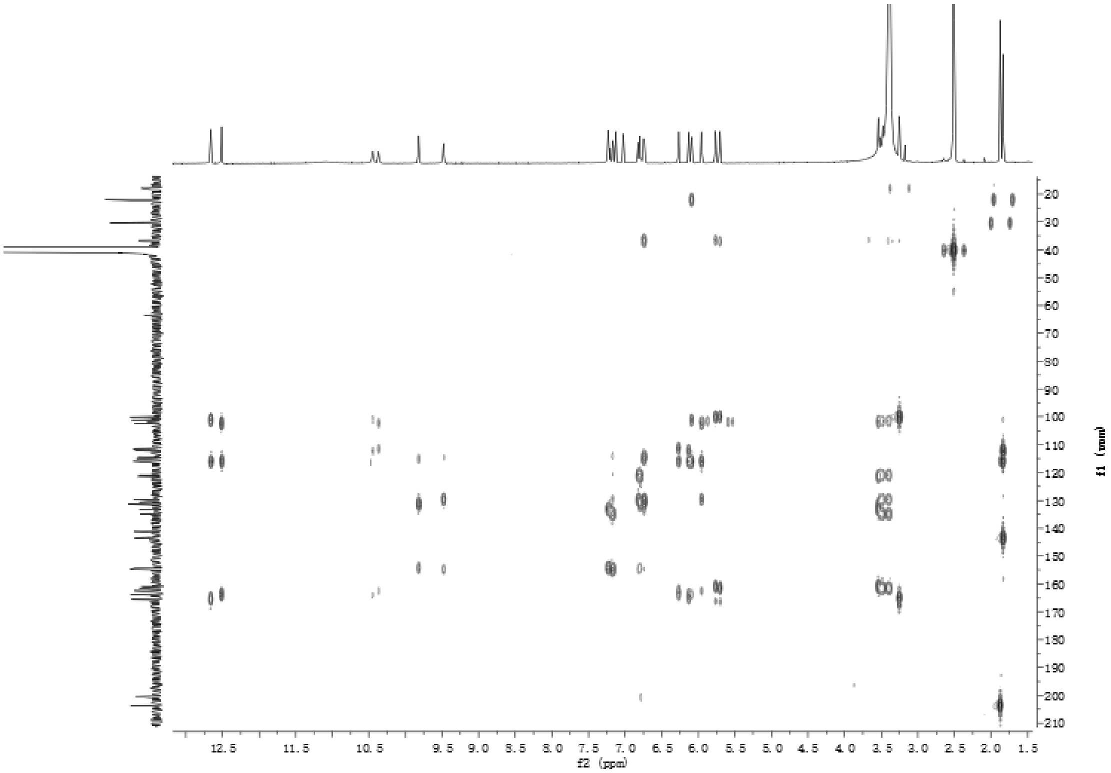

Supplementary Figure 5. HMBC spectrum of **1** (DMSO-d_6, 500 MHz).

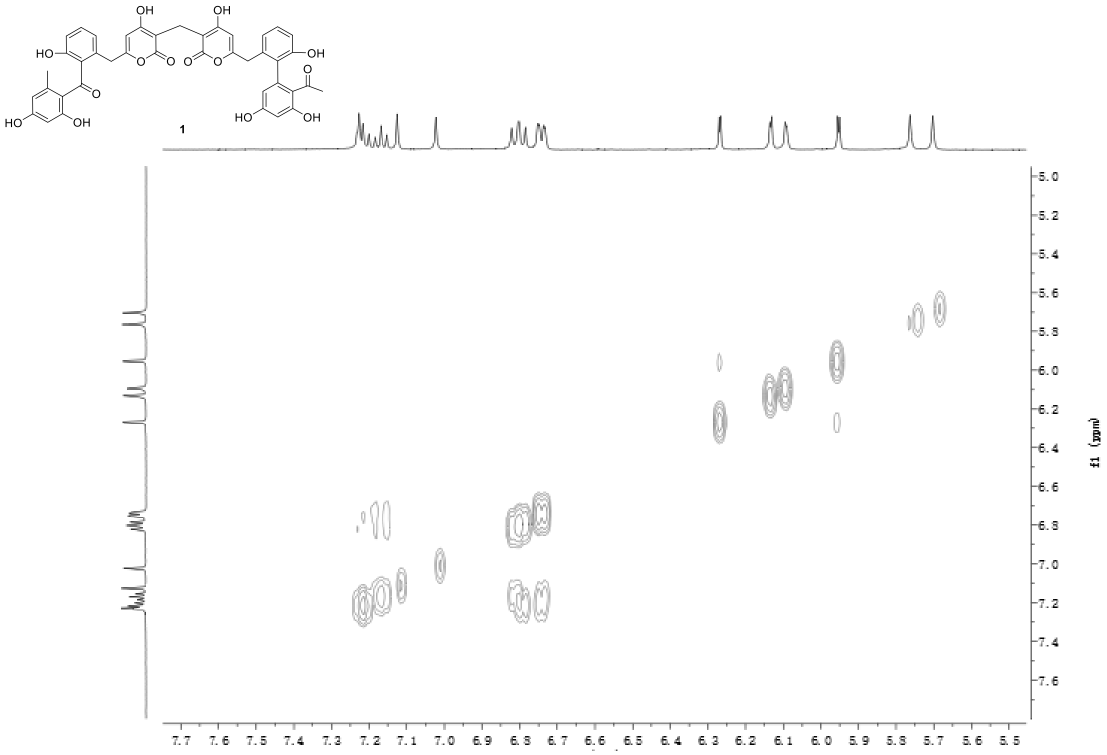

Supplementary Figure 6. ^{1}H-^{1}H COSY spectrum of **1** (DMSO-d_6, 500 MHz).

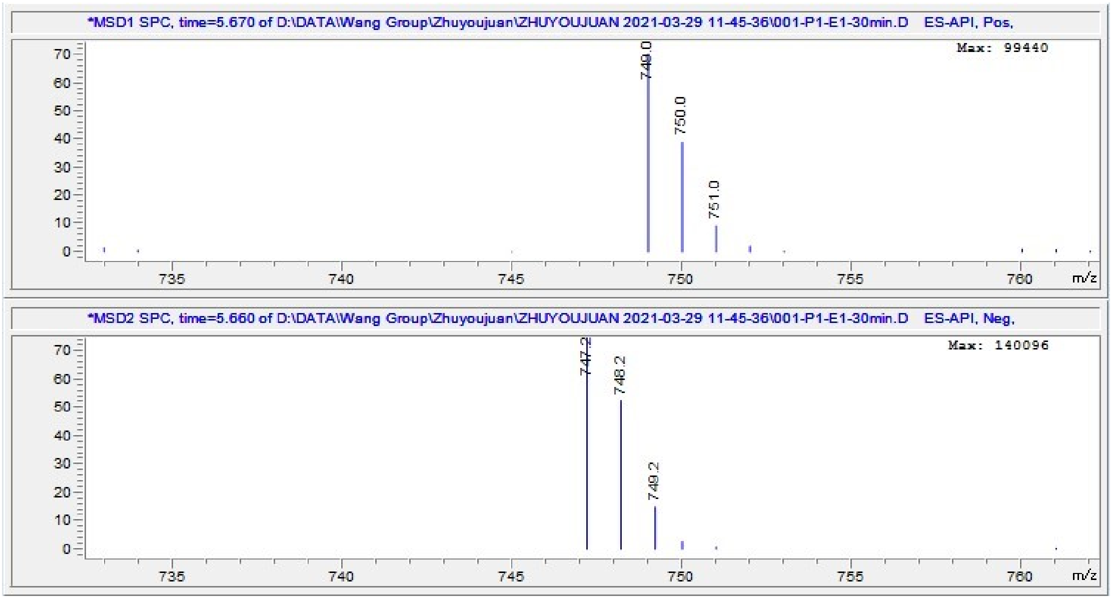

Supplementary Figure 7. (+) and (–)-ESI-MS of **1**.

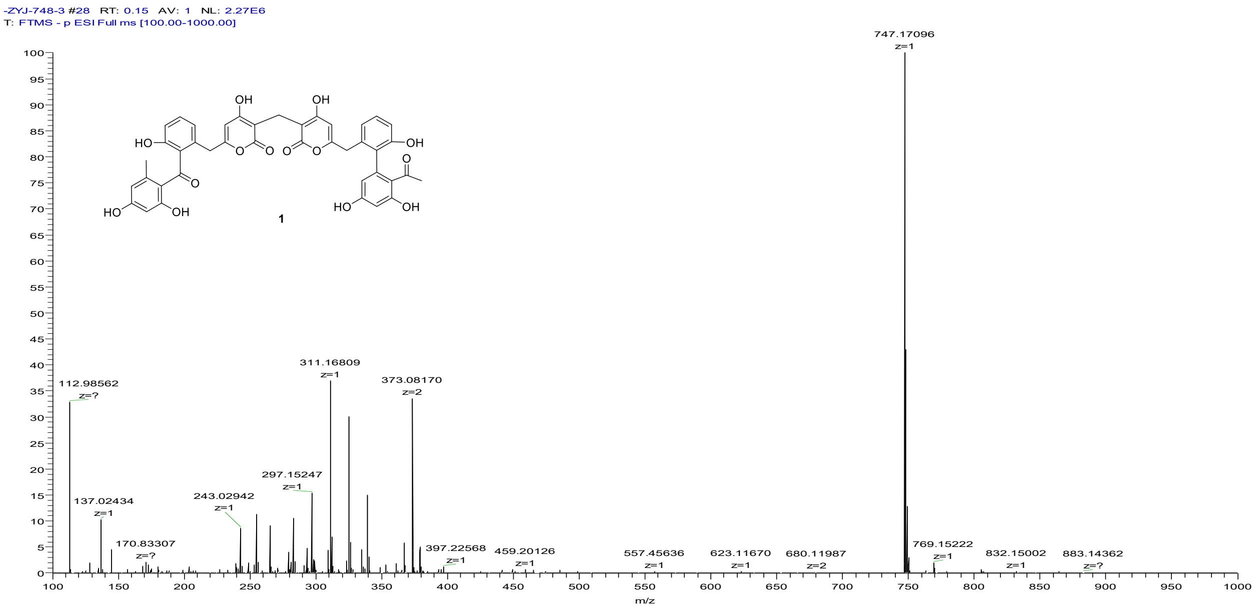

Supplementary Figure 8. HR-ESI-MS spectrum of **1**.

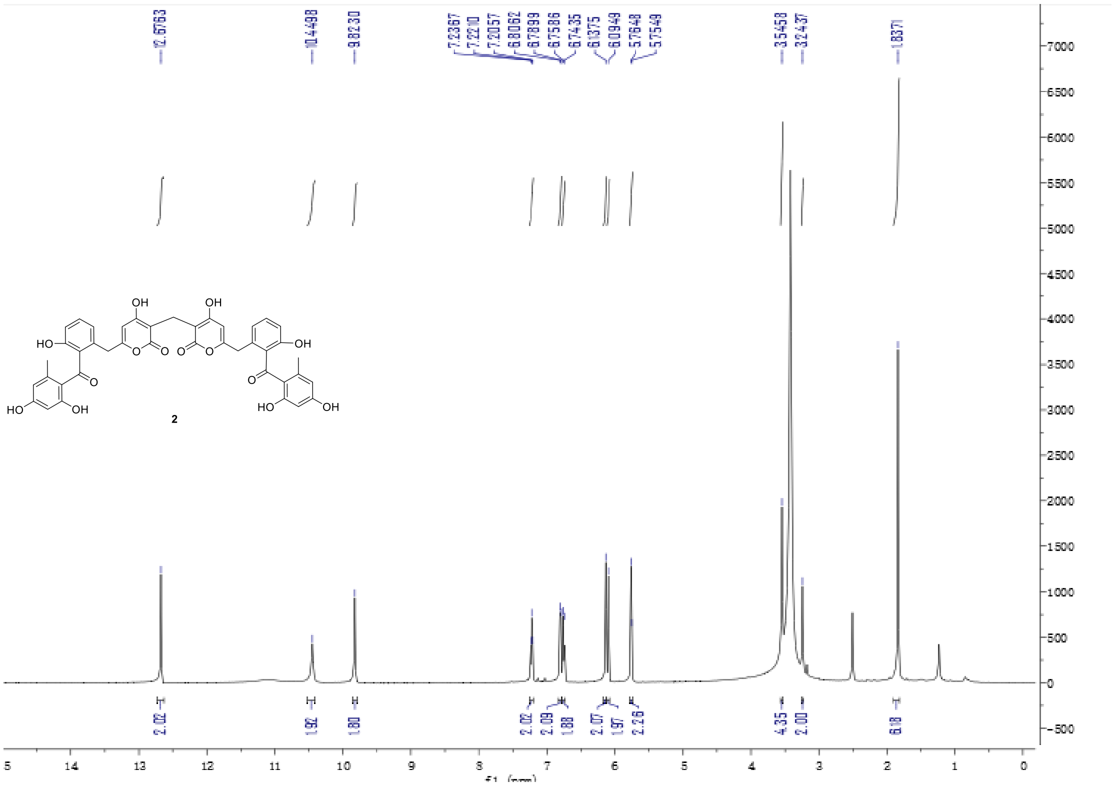

Supplementary Figure 9. ^{1}H NMR spectrum of **2** (DMSO-d_6, 500 MHz).

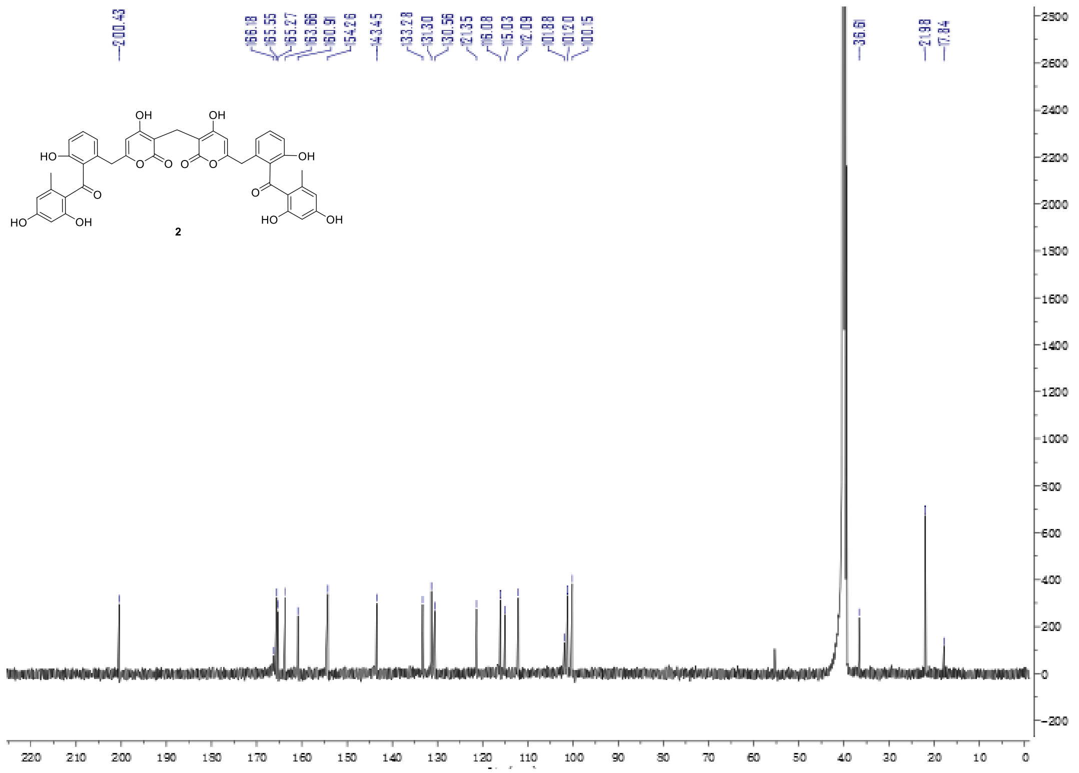

Supplementary Figure 10. ^{13}C NMR spectrum of **2** (DMSO-d_6, 125 MHz).

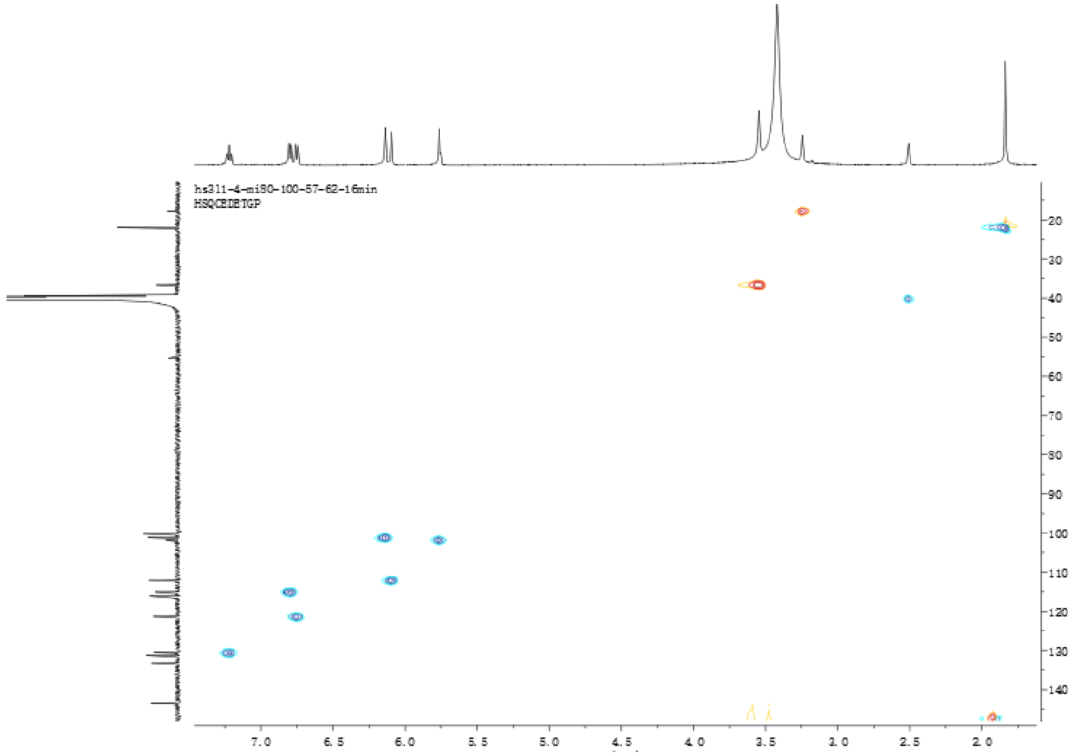

Supplementary Figure 11. HSQC spectrum of **2** (DMSO-d_6, 500 MHz).

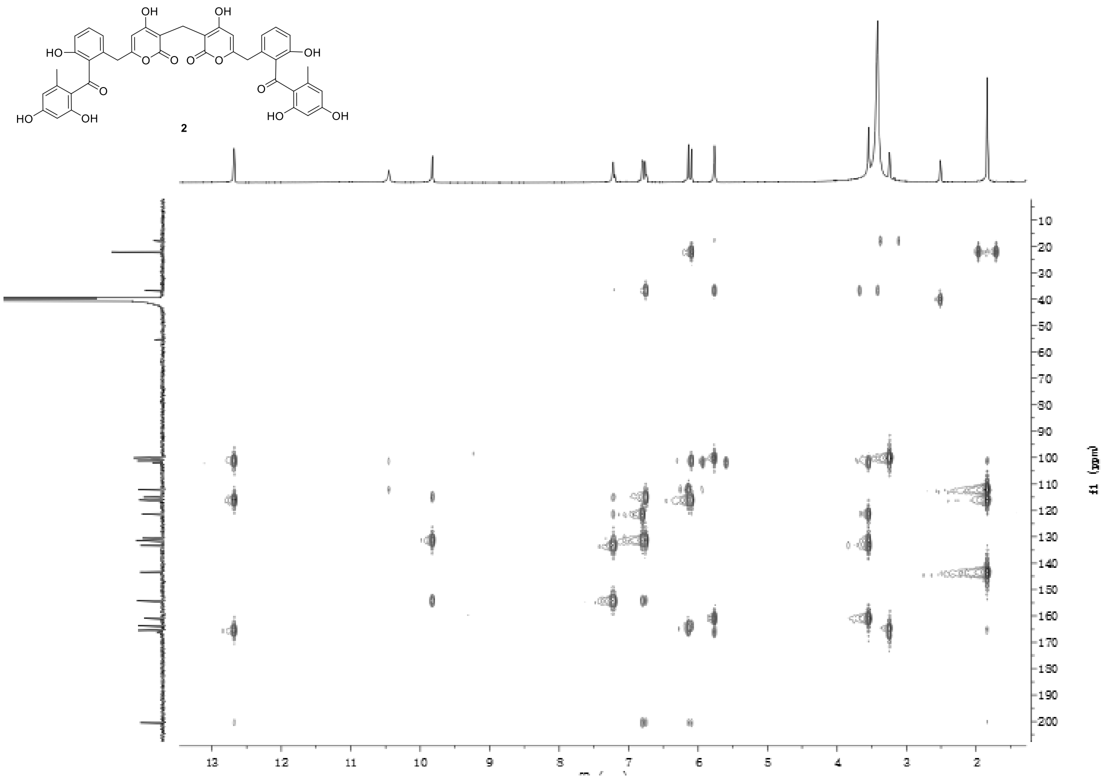

Supplementary Figure 12. HMBC spectrum of **2** (DMSO-d_6, 500 MHz).

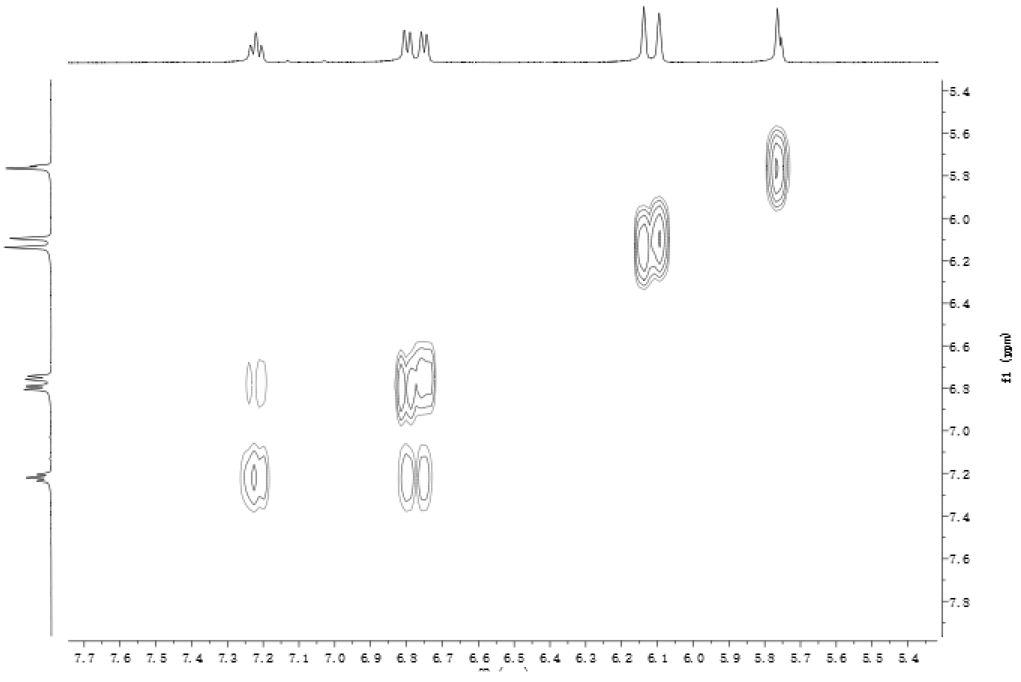

Supplementary Figure 13. ^1H-^1H COSY spectrum of **2** (DMSO-d_6, 500 MHz).

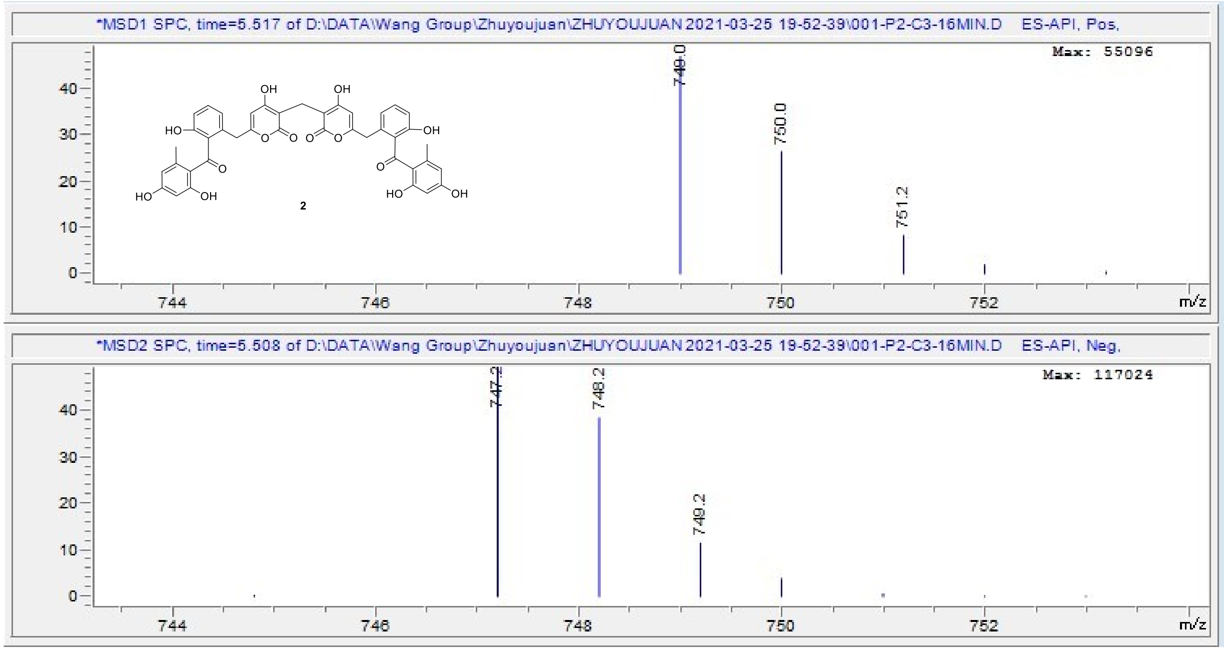

Supplementary Figure 14. (+) and (–)-ESI-MS of **2**.

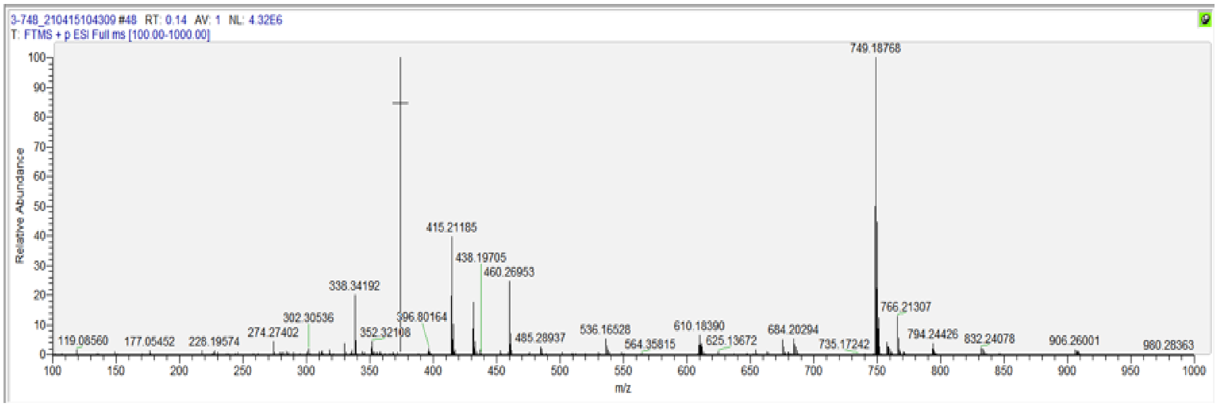

Supplementary Figure 15. HR-ESI-MS spectrum of **2**.

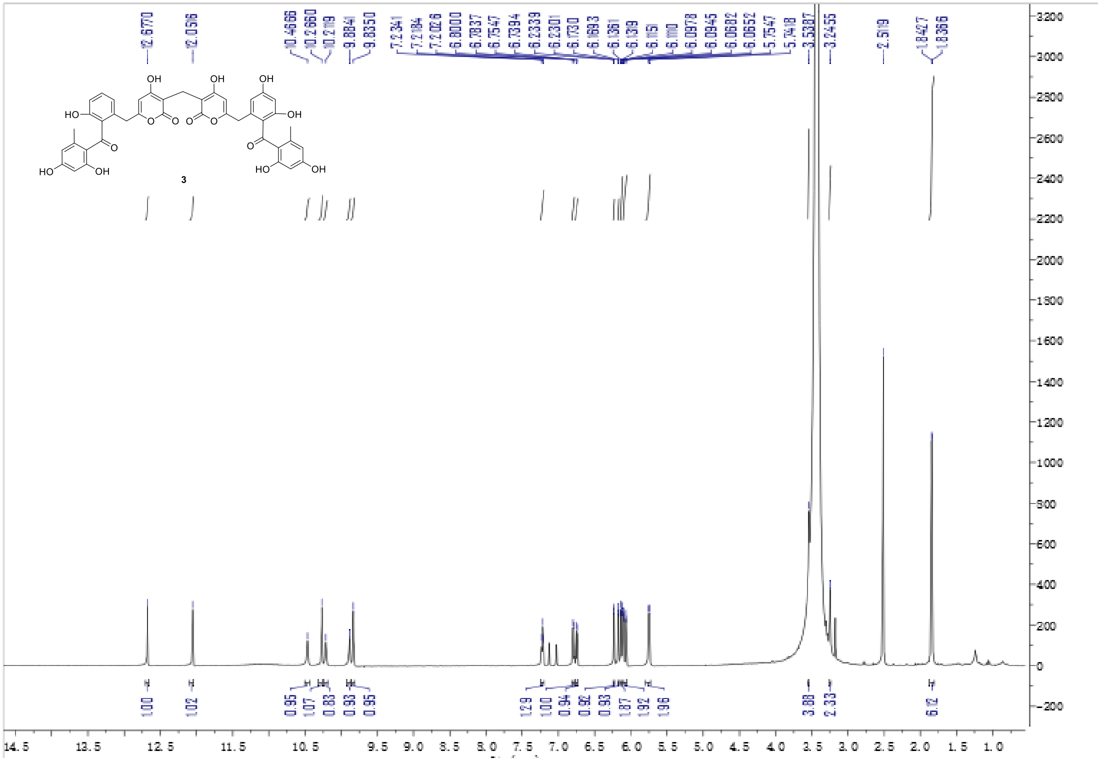

Supplementary Figure 16. ^{1}H NMR spectrum of **3** (DMSO-d_6, 500 MHz).

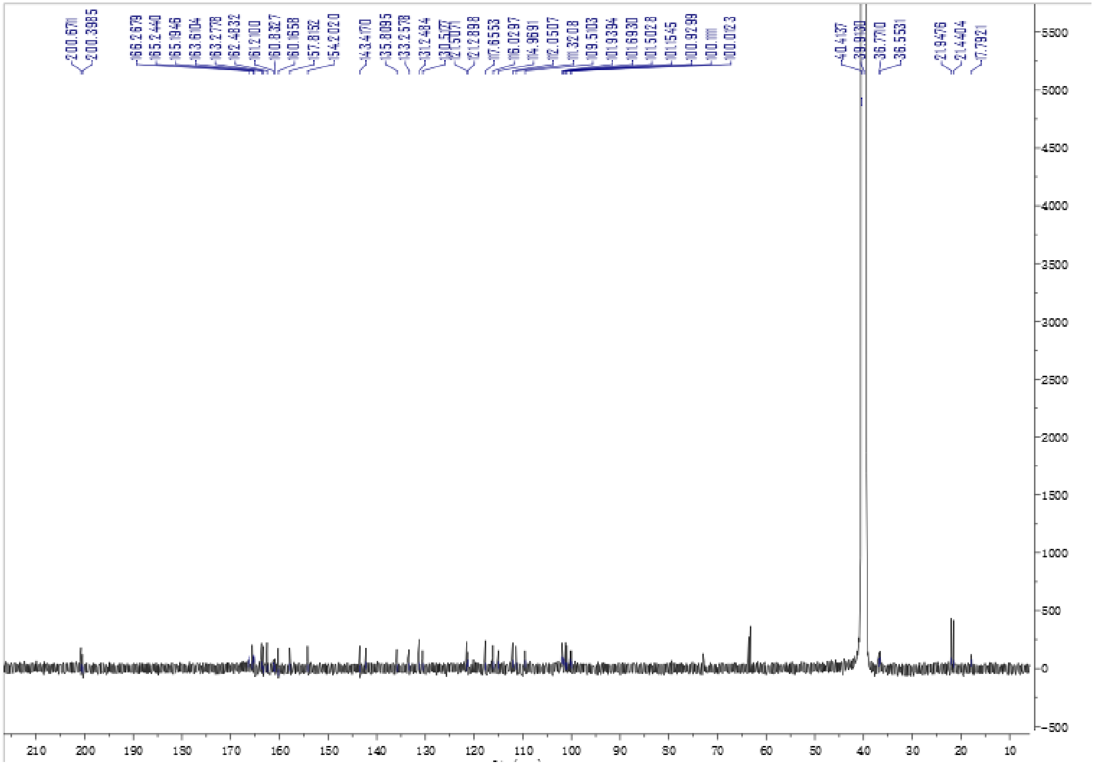

Supplementary Figure 17. ^{13}C NMR spectrum of **3** (DMSO-d_6, 125 MHz).

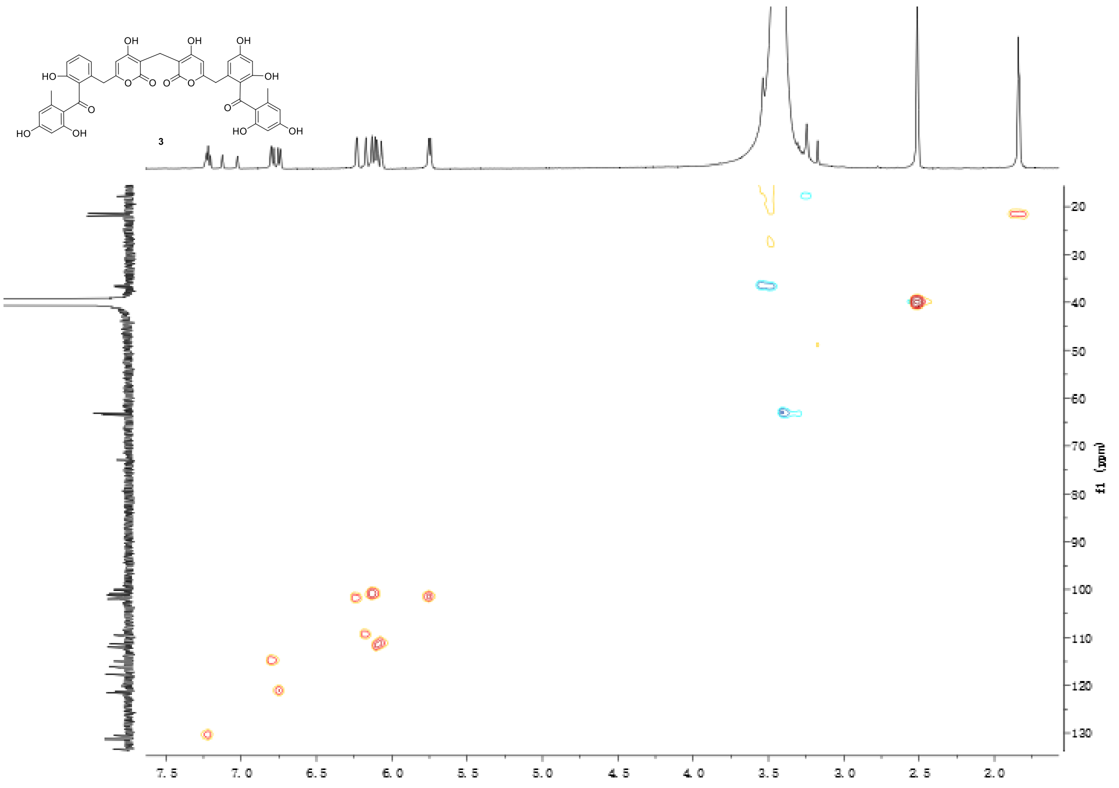

Supplementary Figure 18. HSQC spectrum of **3** (DMSO-d_6, 500 MHz).

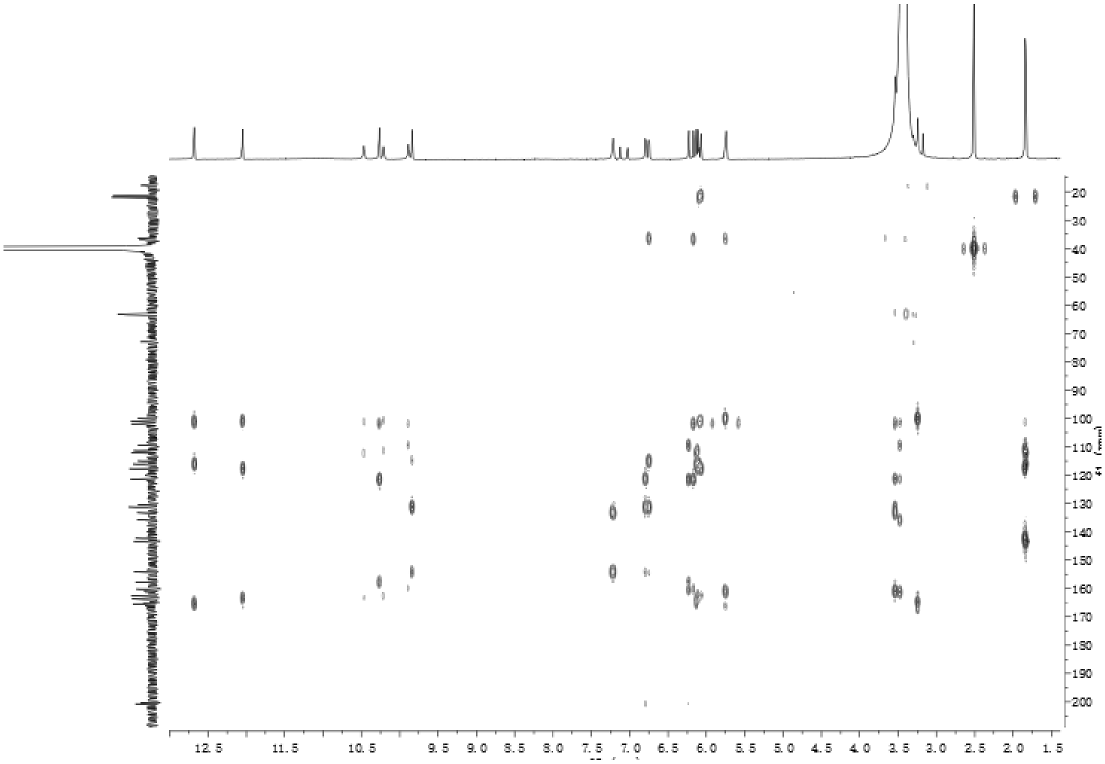

Supplementary Figure 19. HMBC spectrum of **3** (DMSO-d_6, 500 MHz).

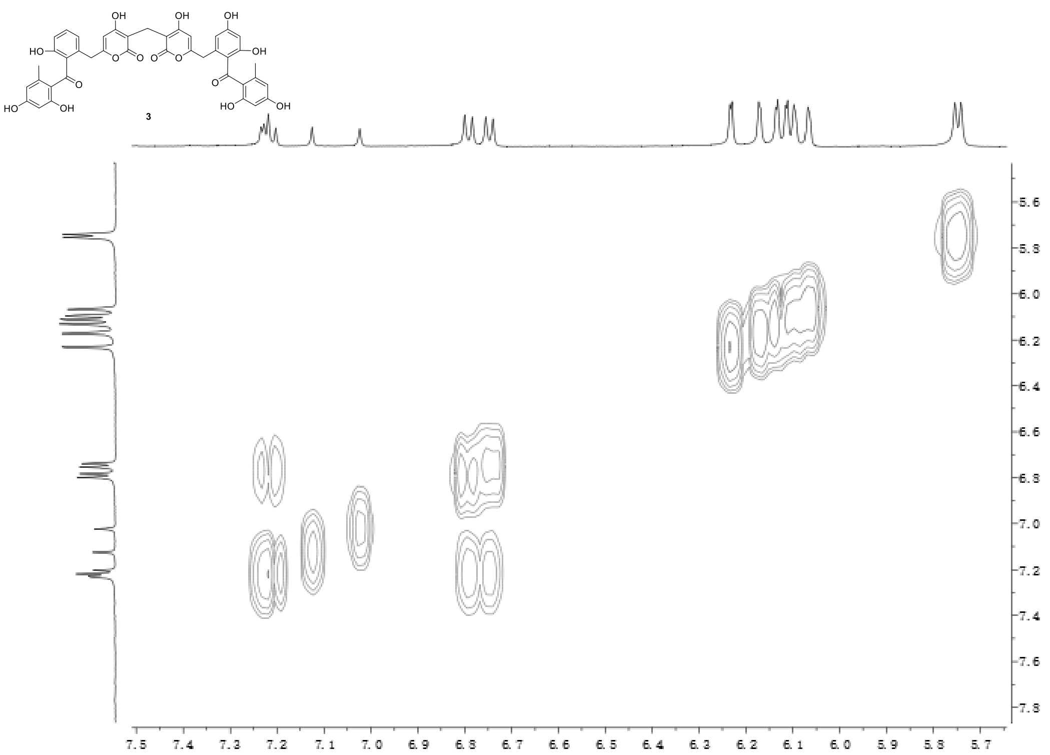

Supplementary Figure 20. ^{1}H-^{1}H COSY spectrum of **3** (DMSO-d_6, 500 MHz).

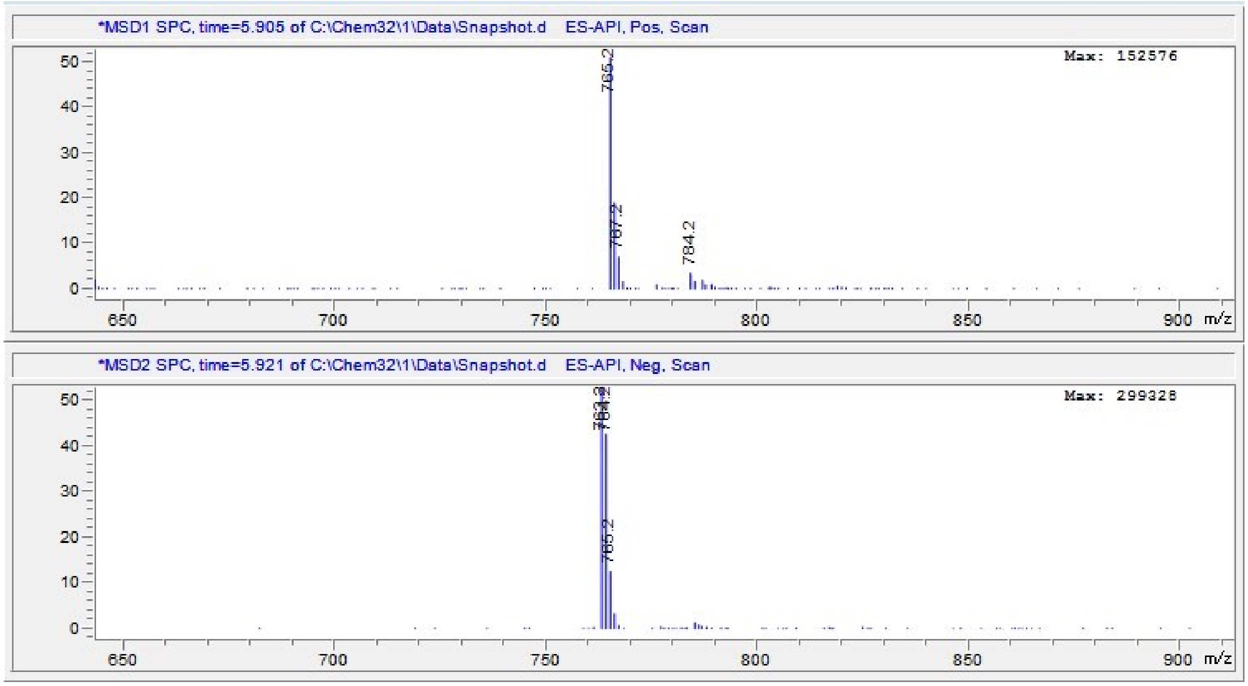

Supplementary Figure 21. (+) and (–)-ESI-MS of **3**.

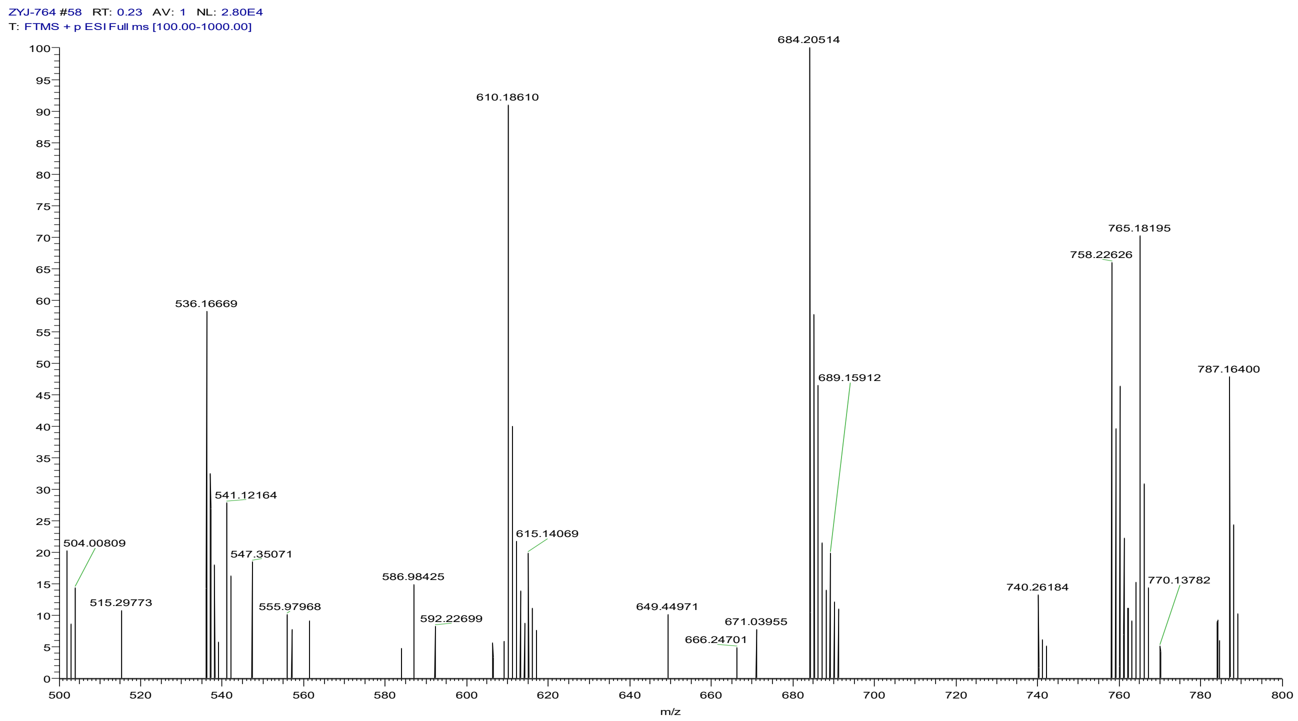

Supplementary Figure 22. HR-ESI-MS spectrum of **3**.
